# Orthographic Learning of Inconsistent Non-Words in Good and Poor Spellers: Linking Dictation and Eye-Tracking Measures

**DOI:** 10.3390/bs16010022

**Published:** 2025-12-22

**Authors:** Julie Robidoux, Antonin Rossier-Bisaillon, Boutheina Jemel, Brigitte Stanké

**Affiliations:** 1 École d’Orthophonie et d’Audiologie, Université de Montréal, 7077, Avenue du Parc, Local 3001-1, Montréal, QC H3N 1X7, Canada; 2 Centre de Recherche Interdisciplinaire en Réadaptation du Montréal Métropolitain (CRIR), 6363, Chemin Hudson, Bureau 061, Montréal, QC H3S 1M9, Canada; 3 Institut Universitaire sur la Réadaptation en Déficience Physique de Montréal (IURDPM), Pavillon Lucie-Bruneau, CIUSSS du Centre-Sud-de-l’Île-de-Montréal, 2275, Avenue Laurier Est, 2^e^ étage, Montréal, QC H2H 2N8, Canada; 4 Laboratoire de Recherche en Neurosciences et Électrophysiologie Cognitive, Hôpital en Santé Mentale Rivière-des-Prairies, CIUSSS du Nord-de-l’Île-de-Montréal, 7070, Boulevard Perras, Montréal, QC H1E 1A4, Canada

**Keywords:** orthographic learning, spelling skills, inconsistencies, eye-tracking, children, French

## Abstract

The French writing system contains numerous phoneme-to-grapheme inconsistencies that vary in their properties and distribution across words. These inconsistencies represent a major challenge for children learning to spell, especially for poor spellers or children with dyslexia-dysorthographia. To our knowledge, no study has examined how inconsistencies shape orthographic learning using both eye-movement data and dictation performance, in children with good and poor spelling skills. In this eye-tracking study, twenty French-speaking children aged 9 to 12 (good spellers: *n* = 10; poor spellers: *n* = 10) learned the spelling of six bisyllabic non-words containing an inconsistent syllable across three learning cycles while we recorded their eye movements. One week later, children completed delayed dictation and recognition tasks assessing long-term consolidation and retrieval. Both groups improved their spelling accuracy and exhibited shorter and fewer fixations across learning cycles, reflecting progressive orthographic learning. However, poor spellers fixated more often and longer on the inconsistent syllable and demonstrated weaker long-term retention, suggesting a less holistic encoding and difficulties consolidating orthographic representations over time. Future research should examine whether these learning patterns generalize to real words, classroom contexts, and to children with dyslexia-dysorthographia across broader learning conditions.

## 1. Introduction

### 1.1. Inconsistencies and Learning to Spell

Alphabetic writing systems, in which sounds are mapped onto written symbols, can be classified along a transparency continuum based on the variability of correspondences between phonemes, i.e., word sounds, and graphemes, i.e., letters or letter clusters used to write words. At one end of this transparency continuum are shallow orthographies such as Spanish, Italian, and Finnish, where most phonemes are represented by a single grapheme. At the other end are deep orthographies such as English, characterized by a greater variability in phoneme-to-grapheme (P-G) mappings (Caravolas, 2004; Fayol, 2009; Seymour et al., 2003). The French writing system falls toward the deeper end of this continuum (Fayol, 2009; Fayol & Jaffré, 2008), with considerable spelling variability: on average, a single French phoneme can be spelled in more than three different ways (Ziegler et al., 1996). This variability creates multiple potential spellings for many sounds, requiring learners to acquire both the specific and general regularities of their writing system (Fayol, 2009; Treiman, 2017).

The degree of variability in P-G correspondences is often referred to as the orthographic “consistency” (Lété et al., 2008). For instance, the phoneme /u/ in French is almost always spelled “ou” while the phoneme /$\tilde{ɛ}$/ may appear as “in”, “ain”, “ein”, etc., depending on the word. Thus, the phoneme /u/ reflects a consistent P-G correspondence whereas the phoneme /$\tilde{ɛ}$/ exemplifies an inconsistent one. However, alternative spellings of inconsistent P-G mappings are not equally distributed in the lexicon. Among the possible spellings of the phoneme /$\tilde{ɛ}$/, the grapheme “in” is far more frequent and regular than the graphemes “ain” and “ein”. In contrast, to spell the phoneme /ã/, there are two frequent graphemes that can be used, “an” and “en”, providing little statistical cues to guide spelling choices (Ruberto et al., 2025). For an estimation of P-G frequencies and regularities in French, see the Manulex-Infra database (Peereman et al., 2007) or grapheme’s classification from Catach (2014). Such disparities in graphemic distribution may influence orthographic learning, as balanced mappings offer weaker probabilistic cues than highly skewed ones, making the selection and consolidation of the correct spelling more demanding for learners. 

Given these inconsistencies, phonological strategies are not sufficient for accurate spelling in French: only about half of all words can be spelled correctly through P–G conversion alone (Véronis, 1988). Unsurprisingly, children face their greatest difficulties with spelling inconsistent words, i.e., with inconsistent P-G correspondences (Ziegler, 2018), which are a frequent source of spelling errors among French-speaking students (Daigle et al., 2016; Hazard et al., 2020; Plisson et al., 2013). Unlike reading, where partial or imprecise orthographic representations may still allow correct recognition, spelling requires complete and exact orthographic traces to be retrieved from long-term memory (Bosman & Van Orden, 1997; Conrad et al., 2018; Perfetti, 1997; Treiman et al., 2025). For inconsistent words, successful spelling entails knowing which specific grapheme maps onto each phoneme among several possible alternatives.

Becoming a proficient speller in French requires acquiring and integrating several complementary types of knowledge (Apel, 2011; Fayol, 2009; Treiman, 2017).

- Phonological knowledge enables the identification and manipulation of phonemes, as well as the mastery of basic P-G correspondences (Fayol, 2009; Sprenger-Charolles et al., 2003).
- Specific orthographic knowledge (or lexical knowledge) corresponds to word-specific orthographic representations stored in long-term memory, enabling rapid and precise access to written forms (Apel, 2011; Apel et al., 2019; Fayol, 2009). The term “orthographic lexicon” refers to the long-term storage of orthographic representations (Buchwald & Rapp, 2009; Fayol & Jaffré, 2024; Rapp et al., 2016).
- General orthographic knowledge reflects sensitivity to broader orthographic patterns, which include graphotactic knowledge, i.e., positional and combinatorial constraints on letters and to permissible grapheme sequences in the written language (Apel et al., 2019; Fayol, 2009; Pacton et al., 2001). Children implicitly acquire these statistical regularities of their writing system well before formal literacy instruction, through repeated exposure to print (Pacton et al., 2001; Treiman, 2017).
- Morphological knowledge allows the identification of morphemes (roots, affixes, grammatical markers) and the use of morphological relationships to determine spellings and maintain silent letters (Casalis & Colé, 2018; Fayol, 2009; Sénéchal, 2000).
- Finally, metalinguistic knowledge involves the ability to consciously apply contextual and morphological rules, and to reason about the functioning of the orthographic system (Pacton et al., 2005).

The literature converges in considering that a competent speller dynamically integrates these different sources of knowledge, developed jointly through exposure to print and explicit instruction (Fayol, 2009).

For inconsistent words, both statistical regularities of the writing system and morphology can help in selecting the appropriate grapheme. Statistical regularities favor frequent and regular P-G correspondences (e.g., selecting “in” instead of “ain” for the phoneme /$\tilde{ɛ}$/) when attempting to spell unfamiliar words (Dutemple, 2023; Fayol, 2009; Kessler et al., 2013; Treiman & Kessler, 2025). Thus, some positional constraints in French restrict which letter sequences are allowed in specific locations (e.g., a consonant cannot be doubled when it is preceded by another consonant) (Pacton et al., 2013; Treiman, 2017; Treiman & Kessler, 2022). It therefore supports spelling by eliminating implausible grapheme choices and favoring letter patterns that conform to the regularities or patterns within the writing system (Fayol, 2009; Pacton & Afonso-Jaco, 2015). Morphological relationships may also provide cues to the conventional spelling of related words (e.g., the silent final *-d* in the word “*grand*” (tall; masculine) revealed by morphologically related words “*grande*” (tall; feminine) and “*grandeur*” (size or height)) (Casalis & Colé, 2018; Sénéchal, 2000; Treiman, 2017). However, these sources of information often remain insufficient, particularly when several spellings are phonologically plausible and permitted by the writing system, as is the case for the phoneme /o/ (e.g., “o”, “au”, or “eau”) or the phoneme /ã/ (e.g., “an” or “en”) in nouns. Furthermore, while the reliance on general orthographic knowledge can facilitate spelling in many cases, it may also result in errors when children apply frequent patterns to inconsistent words.

### 1.2. Theoretical Frameworks of Orthographic Learning

Orthographic learning refers to the process through which orthographic representations are established and refined in memory (Apel et al., 2019; Castles et al., 2018/2018). This process supports both rapid, automated word recognition in reading, and efficient retrieval of conventional spelling during writing. It enables the shift from phonological decoding to direct visual access to word forms during reading and spelling (Castles & Nation, 2006; Castles et al., 2018/2018; Ehri, 2014; Nation & Castles, 2017). Orthographic processing refers to the cognitive mechanisms involved in forming, consolidating, and retrieving orthographic representations in the orthographic lexicon (Apel, 2011; Carroll et al., 2025), and the lexical orthographic memory is the specific memory enabling these processes (Stanké, 2016). To integrate the orthographic lexicon, each new word is encoded as a memory trace and gradually stabilized through repeated exposure until it becomes fully accessible for accurate spelling (Fayol & Jaffré, 2024; Guillery-Girard et al., 2008).

Several models have sought to explain how orthographic representations are acquired, emphasizing the mechanisms that determine the pace and consistency of learning. We will briefly describe three of them: the Self-Teaching Hypothesis, the Integration of multiple patterns model, and the BRAID-Learn model.

#### 1.2.1. Self-Teaching Hypothesis

The Self-Teaching Hypothesis (Share, 1995) is widely used to explain how specific orthographic knowledge develops through reading. The core principle of this model is that each successful phonological decoding of a written word (i.e., associating the correct phoneme to each grapheme of a word) contributes to the formation or strengthening of its specific orthographic representation in memory. With repeated encounters, the link between a word’s phonological and orthographic forms becomes increasingly robust, enabling faster and more automatic recognition. The development of word-specific representations also promotes the generalization of orthographic regularities, thereby supporting the emergence of general orthographic knowledge (Ginestet et al., 2020a; Li & Wang, 2023; Nation & Castles, 2017; Share, 1995). While the Self-Teaching Hypothesis highlights phonological decoding as the primary mechanism driving orthographic learning, the contribution of other cognitive processes, such as statistical learning and visual attention, remains less clearly defined (Ginestet et al., 2020a; Nation & Castles, 2017). Moreover, as Nation and Castles (2017) emphasized, further experimental research is needed to capture orthographic learning as it unfolds in real-time, rather than inferring it only from post-learning outcomes.

#### 1.2.2. Integration of Multiple Patterns Model

The Integration of Multiple Patterns (IMP) model (Treiman, 2017; Treiman & Kessler, 2014) accounts for spelling development beyond phonological knowledge, highlighting how children integrate multiple linguistic cues during spelling acquisition. According to this framework, specific and general orthographic knowledge develop conjointly, as learners draw on previously acquired morphological and statistical regularities within the writing system (Treiman, 2017; Treiman & Kessler, 2022, 2025). Through this implicit and probabilistic learning, also named “statistical learning”, children become increasingly sensitive to the patterns that govern their writing system through incidental exposure to print, even before formal instruction (Apel et al., 2019; Treiman, 2017; Treiman & Kessler, 2014). Orthographic representations are therefore easier to encode and retrieve when they align with these general orthographic regularities, illustrating how orthographic learning integrates phonological, statistical, and morphological information (Treiman, 2017).

#### 1.2.3. BRAID-Learn Model

The BRAID-Learn model (Ginestet et al., 2022) offers a computational account of orthographic learning that integrates visual attentional processing. Complementing Self-Teaching Hypothesis (Share, 1995), it explains how visual attention contributes to the encoding and consolidation of orthographic representations. According to this model, orthographic learning results from the interaction between (1) bottom-up visual input, which is modulated by visual attention and includes visual letter form processing as well as letters’ perception and identification; and (2) top-down lexical feedback from the orthographic lexicon. When a word is novel, visual attention is distributed to maximize letters’ encoding and perception in order to form a new orthographic representation in the lexicon. For instance, visual attention might be greater on graphemes that are less predictable based on familiar orthographic patterns (e.g., the letter “g” in the word “*doigt*” (finger), which is rarely silent in French). With repeated encounters, this representation becomes more stable and more easily activated through top-down processes. Letter identification is then faster, as is word recognition. The BRAID-Learn model therefore illustrates how visual, lexical, and attentional mechanisms jointly support orthographic learning (Ginestet et al., 2022).

#### 1.2.4. Complementarity of the Three Frameworks and Extension to Inconsistencies

Taken together, these frameworks describe complementary mechanisms supporting orthographic learning. The Self-Teaching Hypothesis (Share, 1995) highlights the role of decoding—using P-G conversion to generate and strengthen word-specific orthographic representations through repeated exposure (Ginestet et al., 2020a; Nation & Castles, 2017; Share, 1995). Although the IMP model (Treiman, 2017; Treiman & Kessler, 2014) is primarily a developmental framework, it underscores how statistical learning, sensitivity to patterns and regularities, and morphological relationships contribute to the encoding and retrieval of orthographic forms. The BRAID-Learn model (Ginestet et al., 2022) adds a visual attentional dimension, proposing that attention dynamically adjusts to maximize letter strings processing, with greater attention allocated to letters that are difficult to identify or not predicted by the orthographic lexicon through top-down activation.

Orthographic inconsistencies therefore challenge all these mechanisms. They cannot be resolved by phonological knowledge alone, as several alternative graphemes can represent the same phoneme. Their accurate learning requires the combined support of general orthographic knowledge, morphology, and visual attention, which together contribute to building and stabilizing word-specific orthographic representations through repeated exposure. Inconsistent words likely elicit greater visual attentional resources to ensure accurate encoding, as their orthographic representation may be harder to stabilize in memory given the variability of P-G correspondences. These words may therefore require additional exposures to reach consolidation. In some other situations, only lexical orthographic memory can be mobilized to develop the orthographic representation. This is the case when multiple graphemic options are possible to spell an inconsistent P-G correspondence and where general orthographic knowledge (statistical regularities or morphological relationships) does not uniquely determine the correct spelling. In such situations, orthographic learning necessarily relies on the memorization of word-specific orthographic forms. This study therefore targets these case figures.

### 1.3. Dyslexia-Dysorthographia and Orthographic Learning

While inconsistencies challenge the development of stable orthographic representations, their impact appears particularly pronounced in individuals with dyslexia–dysorthographia (DD)—the French term referring to dyslexia, a specific and persistent neurodevelopmental disorder affecting the acquisition of reading and/or spelling (Carroll et al., 2025). Individuals with DD typically exhibit persistent spelling difficulties and struggle to maintain accurate word spelling in long-term memory. In contrast, their reading difficulties often lessen with age or mainly manifest as slower reading (Carroll et al., 2025; Maughan et al., 2009, 2020; Pugh & Verhoeven, 2018; Snowling et al., 2020).

When the precise orthographic representation of an inconsistent word has not yet been consolidated, learners tend to select another plausible grapheme to compensate for imprecisions in their word-specific representations (Conrad et al., 2018; Perfetti, 1992). In the context of the French writing system, which offers several competing graphemes for the same phoneme, children with DD, poor spellers, and their typically developing peers frequently produce phonologically plausible errors when spelling inconsistent words (Bodard et al., 2023; Daigle et al., 2016; Plisson et al., 2013). This indicates that they rely on the probabilistic characteristics (i.e., frequency, regularity) of French P-G correspondences. They may also draw on morphological knowledge when morphologically related forms are available (e.g., *grand*/*grande*/*grandeur*), sometimes using such cues as a compensatory strategy for their phonological difficulties (Quémart & Casalis, 2017). Nevertheless, morphology alone does not always determine the correct spelling of inconsistent French words. Reliance on partial phonological, probabilistic, or morphological cues may therefore lead to typical error patterns in the presence of inconsistent spellings. French-speaking poor spellers have even been shown to overuse the most frequent grapheme to compensate for their limited word-specific orthographic knowledge (Dutemple, 2023).

Research on orthographic learning also showed that individuals with DD require more time to build complete and stable orthographic representations, and to retain them over time (Binamé et al., 2015; Mehlhase et al., 2019; Poncelet et al., 2003). Poncelet et al. (2003) reported greater forgetting of newly learned spellings after one week in adults with DD. Similarly, Binamé et al. (2015) found that French-speaking children with DD performed lower than their peers both during initial learning and the one-week delayed dictation, suggesting weaknesses in initial encoding and/or long-term consolidation. Mehlhase et al. (2019) observed comparable patterns in German-speaking children: participants with isolated spelling deficits succeeded in building new orthographic representations but struggled to maintain them over time, whereas those with combined reading and spelling deficits showed difficulties at both encoding and retention stages.

In summary, findings from French-speaking populations indicate that individuals with DD or poor spelling skills rely heavily on the most frequent P-G correspondences, on general probabilistic knowledge of the writing system, and on morphology when their word-specific orthographic representations have not been sufficiently consolidated (Bodard et al., 2023; Daigle et al., 2016; Dutemple, 2023; Plisson et al., 2013; Quémart & Casalis, 2017). Yet, consolidation requires substantial and repeated exposure and is particularly demanding for learners with DD as they need more exposures to initially encode the orthographic form and additional practice to maintain these representations in memory (Binamé et al., 2015; Poncelet et al., 2003).

### 1.4. Using Eye-Tracking to Explore Orthographic Learning

Eye-tracking provides a valuable technique to examine orthographic learning as it occurs in real time. Through the analysis of fixations—moments when the gaze remains still—and saccades—rapid eye movements from one word to another—researchers can infer the cognitive processes during reading (e.g., Carter & Luke, 2020; Lai et al., 2013; Rayner, 2009) and, more recently, in orthographic learning (Ginestet et al., 2020b; Joseph & Nation, 2018; Joseph et al., 2014; van Viersen et al., 2022). This technique can provide insights into how partial orthographic representations emerge and gradually evolve across repeated exposures (Nation & Castles, 2017).

Eye-tracking studies have shown that orthographic learning is reflected in systematic changes in eye-movement patterns as exposure increases. Specifically, fixation durations and number of fixations typically decrease, reflecting more efficient visual and orthographic processing as word-specific representations form in memory (Ginestet et al., 2020b; Joseph & Nation, 2018; van Viersen et al., 2022). For instance, Joseph and Nation (2018) asked English-speaking children aged 10 to 11 to silently read sentences containing unfamiliar low-frequency past-tense verbs. Each verb appeared ten times across two sessions, and contextual information was provided to support meaning. Authors found that children read novel verbs more accurately and processed these more quickly after repeated encounters, illustrated by a decrease in total reading time (the sum of the duration of all fixations made on a verb). Children later spelled words more correctly than a control group who was not exposed to these novel verbs. Their findings suggest that children developed stable orthographic representations for the newly learned verbs, as reflected in both their eye-movement patterns and their improved spelling performance (Joseph & Nation, 2018).

Similarly, van Viersen et al. (2022) observed steeper decreases in gaze duration (the sum of the duration of all fixations before the eyes left the word) and total reading time (which include durations during re-viewing of words) across exposures, in Dutch-speaking children from Grades 2 and 5. They manipulated both exposure (two vs. six encounters) and lexicality (real words vs. irregular pseudo-words), embedded in sentences that provided limited semantic context. Overall, younger children showed longer gaze durations and total reading times than older children, and pseudo-words were associated with longer fixations than real words. The decrease in eye-movement measures reached an asymptote around the third and fourth encounters, indicating a rapid gain in familiarity with words during the initial exposures. Eye-movement measures then decreased smoothly during the last exposures. For older children, the decrease in gaze duration and total reading time was even steeper for pseudo-words, suggesting greater effort in forming new orthographic representations than for orally familiar real words (van Viersen et al., 2022). 

Unlike the previous studies conducted with children (Joseph & Nation, 2018; van Viersen et al., 2022), Ginestet et al. (2020b) observed decreases in both total fixation duration and number of fixations across exposures (one, three, or five encounters), in French-speaking adults reading aloud pseudo-words presented in isolation. The steepest decline in eye-movement measures occurred between the second or third presentations, indicating faster visual processing for items appearing more often. In post-test tasks (unexpected dictation and orthographic decision tasks), pseudo-words encountered five times were spelled more accurately and recognized more quickly than those seen once (Ginestet et al., 2020b).

There are at least two limitations to the previously detailed literature on orthographic learning and eye-tracking. First, previous studies measured orthographic learning performance (i.e., behavioral measures such as dictation, recognition task, orthographic decision task) only at the end of the learning task or after the final exposure (see Ginestet et al., 2020b; Joseph & Nation, 2018; van Viersen et al., 2022). However, no study evaluated orthographic representations after each exposure, which would have provided a finer-grained understanding of how visual processing relates to learning outcomes. Furthermore, repeated practice of spelling contributes to consolidating and establishing stronger orthographic representations in the orthographic lexicon (Binamé & Poncelet, 2016; Conrad et al., 2018; Fayol & Jaffré, 2008; Manesse & Cogis, 2007; Ouellette, 2010; Perfetti, 1997). Examining orthographic learning in an explicit learning context, where participants are told to memorize the orthographic form of words, would enable such outcome measurements after each exposure, unlike an incidental reading paradigm.

Second, while the effect of inconsistencies has been documented through behavioral measures (e.g., spelling accuracy and/or error analysis in dictations or texts; Bodard et al., 2023; Daigle et al., 2016; Hazard et al., 2020; Plisson et al., 2013), previous eye-tracking research has yet to provide a fine-grained analysis of visual attention allocation to inconsistencies during orthographic learning. Although words or pseudo-words in prior eye-tracking studies often contained inconsistencies or were irregular (Ginestet et al., 2020b; van Viersen et al., 2022) to prevent strict reliance on P-G mappings, no study has specifically analyzed fixation patterns on inconsistencies to examine how learners process these challenging orthographic features during encoding. For instance, as inconsistencies represent a major difficulty in learning the spelling of words, these might necessitate multiple viewings and longer processing times until the orthographic representation becomes integrated, complete, and precise. Eye-tracking measures could reveal whether and how learners allocate differential attention to inconsistencies, providing insights into the online encoding strategies that support or hinder the consolidation of accurate orthographic representations.

### 1.5. Aim of the Study and Hypotheses

This study aimed to examine how children with good and poor spelling skills learn new orthographic representations of inconsistent non-words. Inconsistencies were related to two different phonemes, with plausible P-G correspondences varying in frequency and regularity. Dictation scores and eye-tracking measures were combined to capture learning progression in real time, and long-term retention was assessed one week later.

To better understand the mechanisms underlying orthographic learning, the present study used spelling performance as a proxy for spelling skills rather than directly comparing children with and without DD. Two main objectives were addressed:

1. To analyze learning progression—from the encoding of the orthographic representation to its retrieval in long-term memory after one week—by combining changes in spelling accuracy and eye-movement measures across repeated exposures.
2. To examine the influence of inconsistencies on visual exploration of non-words during encoding process.

Guided by these objectives and prior findings, we formulated the following hypotheses:

1. Across learning cycles, both groups were expected to learn the spelling of non-words. However, poor spellers were predicted to obtain lower dictation scores in the first learning cycle and to reach the performance of their peers by the final exposure (Binamé et al., 2015; Mehlhase et al., 2019). Both groups were also expected to show a decrease in fixation durations and number of fixations across exposures, reflecting progressive orthographic learning (Joseph & Nation, 2018; van Viersen et al., 2022). For long-term retrieval, good spellers were anticipated to show a slight decrease in their performance after a one-week delay, as typically observed, whereas poor spellers were expected to exhibit a larger decline (Binamé et al., 2015; Mehlhase et al., 2019).
2. During the visual exploration of non-words, both groups were predicted to fixate more often and for longer durations on the inconsistency in order to learn the spelling (Ginestet et al., 2022). Since inconsistencies represent a challenge in spelling, they might require multiple fixations before orthographic representations become well-specified.

## 2. Methods

### 2.1. Participants

Twenty French-speaking children aged 9 to 12 participated in this study (*M*_age_ = 10 years and 9 months, *SD*_age_ = 12 months, 6 boys and 14 girls, mostly at the end of elementary school (grades 5 and 6; 12 out of 20)). They were from Greater Montreal and its surrounding areas. They had normal or corrected-to-normal vision, and none presented with moderate to severe developmental language disorder, deafness, autism spectrum disorder, or intellectual disability. Children with attentional difficulties or attention deficit hyperactivity disorder (ADHD) were nevertheless included, given the well-documented comorbidity between ADHD and DD (Carroll et al., 2025; McGrath et al., 2011; Peterson & Pennington, 2015).

Seven children had received a prior diagnosis or a hypothesis of DD. Parents of these children consistently reported persistent reading and spelling difficulties. Of the remaining 13 children, 11 had no reported history of reading or spelling difficulties, and two had experienced earlier difficulties that parents indicated had since resolved.

Reading and spelling competencies were assessed using word (regular and irregular) and non-word reading and dictation tasks from the *Batterie Analytique du Langage Écrit* (Analytical test battery of written language) (BALE; Jacquier-Roux et al., 2010). Spelling skills did not align strictly with diagnostic status: some children with DD performed well on both word and non-word dictations, while some with no history of spelling difficulties obtained lower scores. To account for this heterogeneity, the sample was reclassified according to dictation performance. Specifically, the irregular word score was used as these words must be memorized for accurate spelling and thus provide an index of word-specific orthographic knowledge. Children scoring above the median were assigned to the good spellers group *(n* = 10), and those scoring below the median to the poor spellers group (*n* = 10). Children school grades’ distribution was slightly different across group, with half of poor spellers in grades 3 and 4, against 3 out of 10 in good spellers. Nevertheless, for descriptive purposes, participant scores were compared to the BALE normative data for their grade level (Jacquier-Roux et al., 2010). Due to differences in test administration, normative data could not be used initially to form the groups. Poor spellers mostly scored near or below the 25th percentile, whereas good spellers mostly scored above the 50th percentile when compared to their grade level. Therefore, even if poor spellers seemed a little younger, their spelling score was still lower according to normative data.

Phonological awareness, working memory and visual attention span were assessed, respectively, with Initial and Final phoneme deletion (BALE; Jacquier-Roux et al., 2010), Digit span test (CELF CDN-F; Semel et al., 2009) and 5-letter global and partial report tasks from Evadys (Valdois et al., 2017a). For the visual attention span, the number of letters accurately reported in both conditions was combined and transformed into percentage, following the procedure by Ginestet et al. (2020b).

Table 1 presents group characteristics and performance across reading, spelling, phonological awareness, working memory and visual attention span tasks. As good spellers were expected to perform better than poor spellers to tasks, Welch’s one-tailed *t*-tests (JASP Team, 2024) were used. No significant group differences were found for age, non-word dictation, initial and final phoneme deletion, backward digit span, and visual attention span. As predicted, poor spellers performed worse than good spellers in regular and irregular word dictations. They also exhibited lower accuracy and slower speed in all reading subtests as well as a lower score in the forward digit span task.

### 2.2. Stimuli of the Orthographic Learning Task

Stimuli were six bisyllabic non-words containing an inconsistency drawn from a standardized clinical assessment tool (*Test de mémoire lexicale orthographique* [Lexical Orthographic Memory Test]; in preparation). The task was designed to assess lexical orthographic memory and its three processes (encoding, storage, and retrieval). Table 2 lists the non-words and their characteristics (frequency and regularity).

For three non-words, the inconsistency involved the French phoneme /ã/ (e.g., *parent* (parent): /paʁ**ã**/), spelled “en” in two non-words, and “an” in one non-word. As indicated in Table 2, frequency and regularity of both P-G correspondences are close. Therefore, it is very hard to choose the right grapheme without knowing the right spelling of a word (Catach, 2014; Ruberto et al., 2025). In two non-words containing this phoneme, the inconsistency occurred in the first syllable, and in the third, it occurred in the second syllable, that is at the end of the non-word. All /ã/ non-words were five letters long.

The three other non-words contained the French phoneme /$\tilde{ɛ}$/ (e.g., *lapin* (rabbit): /lap**$\tilde{ɛ}$**/), with the inconsistency always located in the second syllable, at the end of non-words. Each /$\tilde{ɛ}$/ non-word used a different spelling, resulting in three variants: “ain”, “ein” and “yn”. Importantly, the most frequent (frequency: 9399.51) and regular (regularity: 42.16) grapheme “in” (see Manulex-Infra database; Peereman et al., 2007) was deliberately excluded from the stimuli. This choice aimed at avoiding ceiling effects and to ensure that learning required the acquisition of less frequent graphemes. 

The six non-words were read aloud and recorded by a male native speaker of French in a sound-attenuated room. Recordings were made using a RØDE NTG1 directional microphone (RØDE, Sydney, Australia) connected to an ASIO-compatible sound card (SoundBlaster Audigy 2-ZS, Creative Technology, Singapore) at a 24 kHz sampling rate and 16-bit resolution. Each non-word was read in isolation with neutral prosody and a consistent speech rate to minimize coarticulatory effects. The recordings were segmented into individual audio files corresponding to each non-word using Adobe Audition (version 13.0.7). Files were cleaned of any background or environmental noise, trimmed to remove leading and trailing silences, and normalized in amplitude to ensure consistent intensity levels across non-words. This preprocessing ensured that all auditory stimuli were comparable in clarity, duration, and loudness.

Furthermore, each non-word was paired with a photograph of a familiar real object or animal which provides a referential meaning, thereby facilitating the retention of the orthographic representation (Marinelli et al., 2020; Ouellette, 2010; Ricketts et al., 2009; Wang et al., 2013). We used pictures of real objects rather than invented referents to ensure that children could immediately access a semantic anchor, in addition to the phonological form with the recordings. The task thus focused specifically on the visual–orthographic characteristics of the non-words and reduced the likelihood that learning outcomes reflected decoding or semantic acquisition instead of orthographic encoding.

To ensure copyright compliance, photographs were taken by the research team when possible (3 images: comb, fork, couch) or selected from the Bank of Standardized Stimuli (BOSS; Brodeur et al., 2014) (2 images: squirrel, apricot), which provides high-resolution, normed visual stimuli with standardized ratings of familiarity and name agreement. Photographs were consistent with images used in the original assessment tool. The sixth image, representing a river, was obtained from Pixabay (https://pixabay.com/fr/photos/le-caucase-russie-elbrus-rivi%c3%a8re-5302236/, accessed on 17 June 2021) as no suitable photograph was available in the BOSS database.

### 2.3. Procedure

The overall procedure proposed in the assessment tool was followed. Children learned the spelling of the six non-words across three consecutive learning cycles that included an encoding phase (hereafter referred to as the learning phase) and a dictation phase assessing short-term retention. One week later (7–9 days after the first session), a delayed recall phase assessed retrieval in lexical orthographic memory through a dictation and a recognition task. The delayed recall phase took place either in the laboratory or online (via Zoom), without eye-tracking.

At the beginning of the experimental session, children were first introduced to the task using an example. They were told that their goal was to learn the spelling of words (their orthographic form) in a foreign language. To succeed, they needed to pay close attention to the spelling since they would be later asked to complete a dictation. The experimenter acknowledged that the task might seem challenging and reassured the children that each word would be presented three times, giving them enough time to learn the spelling.

**Figure 1:**
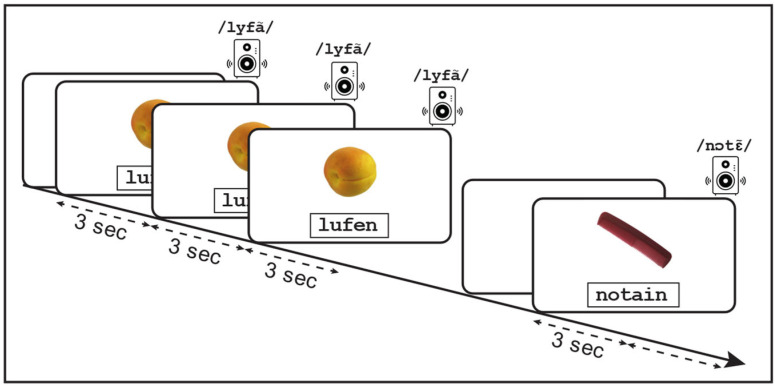
Illustration of a trial during the learning phase, involving the presentation of the orthographic form of a non-word (e.g., “lufen”) alongside its corresponding image (apricot), and the audio pronunciation of the non-word (/lyfã/).

During each learning phase (Figure 1), each non-word and its corresponding image were displayed simultaneously on the screen while the non-word pronunciation was played through speakers. The non-word was read aloud to children to limit the impact of reading skills on encoding, so that the correct phonological form would be encoded. This allowed children to focus solely on learning the orthographic form of the non-word. During each trial, the visual display remained on the screen for 9 s, while the audio pronunciation was played three times—once every 3 s. Non-words were presented in lowercase Courier New, bold, black font, at 60 pixels. Five-letter non-words subtended approximately 4.15° of visual angle horizontally, and six-letter non-words about 4.98° of visual angle. The non-word appeared in the lower half of the screen, and the image in the upper half, both horizontally centered, consistent with the layout in the original assessment tool. Eye movements were recorded throughout the learning phase.

Once all six non-words were presented, the dictation phase began. On each trial of this dictation phase, the image associated with the target non-word appeared on the screen and its pronunciation was played once (repeated on request). Children were then asked to write down the spelling of each non-word by hand on an 8½ × 11-inch sheet of paper displaying the image associated with the target non-word and a blank box underneath. No direct feedback was provided by the experimenter during the dictation phase. However, children received implicit feedback when the non-words were presented to them during the subsequent learning phase.

Learning and dictation phases were repeated twice more, for a total of three learning–dictation cycles. The order of non-word presentation differed across cycles but was identical across participants, in line with the original assessment tool.

A week after completing the three learning cycles, children participated in the delayed recall phase, conducted either in the laboratory or via Zoom. This phase assessed long-term retention of the spelling through a dictation task followed by a recognition task. Children were instructed to recall, as best as they could, the spellings of the non-words they had learned the previous week. The dictation task followed the same procedure as in the learning cycles. For children tested online, the 8½ × 11-inch sheet displaying the image with a blank box underneath was shown on the computer screen. The experimenter provided the pronunciation of each non-word orally, as automatic audio playback was not possible in the online setting. Children were then asked to type the corresponding orthographic form of each non-word. In the recognition task, four alternative spellings were presented on a single line for each non-word, and children had to select the correct one after hearing the experimenter pronounce it. All choices were phonologically plausible given the non-words’ pronunciation. The fill-in choices included grapheme errors on the target P-G correspondences (e.g., “tenvo” vs. “tanvo”; “notain” vs. “notein”), silent letters (e.g., “tenvo” vs. “tenvot”), and consonant doubling (e.g., “notain” vs. “nottain”).

### 2.4. Apparatus

Participants were seated in a quiet room of the lab with lights on, in the presence of one or two experimenters. Eye-tracking data were collected via an EyeLink 1000 Plus system (SR Research, Kanata, ON, Canada) at a 1000 Hz sampling rate. Non-words and their corresponding images were displayed on a computer monitor (Asus, VG248, 1920 × 1080 pixels resolution, 60 Hz refresh rate; Asus, Fremont, CA, United States), and the pronunciation of non-words was played through speakers (M-Audio Bx4, 4.5″, two speakers [left and right]; M-Audio, Cumberland, RI, USA), with the volume level set to a normal conversation level. Stimulus presentation was controlled using Experiment Builder (version 2.3.38; SR Research Ltd., 2020b).

The desktop mount mode with the chinrest (head-stabilized) was used for few participants at the beginning of the data collection. However, due to noticeable data drifts, the remote mode was subsequently adopted to reduce these issues. In both setups, children were seated approximately 96 cm from the screen. The eye-tracking system was calibrated using a standard 9-point calibration and validation procedure was performed at the beginning of each learning phase, with accuracy thresholds set at an average gaze error ≤ 0.5° and a maximum error per position ≤ 1°. A drift check was conducted before the presentation of each non-word on the screen to ensure calibration stability throughout the session.

### 2.5. Analyses

#### 2.5.1. Eye-Movement Data Cleaning

Before analyzing fixation data, several preprocessing steps were performed using Data Viewer (version 4.1.211; SR Research Ltd., 2020a). First, all trials were visually inspected to identify potential vertical drifts. When necessary, fixations located near the area of interest corresponding to the non-words were manually adjusted vertically to correct for inter-trial head movements. Following visual inspection, data cleaning was conducted using the “4-Stage Fixation Cleaning” function in Data Viewer. Only two stages were applied to minimize data loss: (1) merging fixations shorter than 80 ms with an adjacent longer fixation within 0.5° of visual angle, and (2) applying duration thresholds of 80 ms (minimum) and 2000 ms (maximum). The 80 ms lower limit is consistent with previous reading studies (e.g., Eskenazi, 2024) and prior research on orthographic learning (e.g., Joseph & Nation, 2018; van Viersen et al., 2022). The 2000 ms upper limit corresponds to a rounded value of the highest inter-quartile range (3 × [Q3 − Q1]) calculated per participant across the three learning phases (maximum observed: 1990 ms). Overall, 4.17%, 4.93%, and 7.02% of fixations were removed in Learning Phases 1, 2, and 3, respectively.

#### 2.5.2. Data Scoring and Preprocessing

Behavioral data were collected through dictation tasks administered at the end of each of the three learning phases (testing phases 1 to 3) and one week later (testing phase 4), as well as through a recognition task administered one week later (testing phase 5). Non-word spelling was scored as correct (coded as 1) when it exactly matched the orthographic form of the learned non-word. Any deviation from the target spelling was coded as incorrect (coded as 0).

In addition, we documented the specific spellings produced when errors occurred. These analyses were based solely on data from the dictation task, which allowed us to quantify the frequency of each spelling error type separately for non-words containing the phonemes /ã/ and /$\tilde{ɛ}$/.

For the phoneme /ã/, errors consisted of:

- “in” or “an” produced instead of “en”; and
- “en” produced instead of “an”.

For /$\tilde{ɛ}$/, errors included:

- “in”, “ein”, or “yn” for the grapheme “ain”;
- “in”, “en”, “ain”, or “yn” for the grapheme “ein”; and
- “in”, “ein”, or “ain” for the grapheme “yn”.

Rare and atypical spellings (e.g., “ayn”, “ien”) were categorized as “other”. This “other” category also included the very few errors that were not related to the inconsistency (e.g., adding a silent letter).

Eye-tracking data were preprocessed by removing fixations whose durations exceeded three standard deviations from each participant’s mean, as these were considered outliers. In total, 2.27%, 2.09%, and 2.10% of fixations were excluded from the first, second, and third learning phase, respectively. The remaining dataset comprised 2582 fixations for the first learning phase, 2436 for the second learning phase, and 2242 for the third learning phase.

Cleaned fixation data were then reorganized by syllable to examine the effects of group, syllable, and learning phase on fixation duration and number of fixations. For each non-word, the total fixation duration and number of fixations per syllable were the dependent variables. Because syllable length varied (two to four letters), both fixation durations and number of fixations were weighted by letter, for each syllable individually, to avoid a length effect. To do so, eye-movement measures were divided by the number of letters per syllable. For instance, in the non-word “lufen”, fixation measures were divided by 2 for the first syllable (“lu”) and by 3 for the second syllable (“fen”). In the non-word “notain”, they were divided by 2 for “no” and by 4 for “tain”. These normalized measures represented, in the following analyses, the mean fixation duration and the mean number of fixations per syllable. Finally, syllables were recoded as “less consistent/inconsistent syllable”, for the syllable containing the inconsistency, and “more consistent/consistent syllable”, for the one without the inconsistency. To lighten the text and figures, the terms “inconsistent” and “consistent” were preferred, although the consistency of P-G correspondences is better expressed as a continuum.

#### 2.5.3. Statistical Analyses

All statistical analyses were performed in R (R Core Team, 2023) using RStudio (version 2024.12.0). Spelling accuracy (accuracy score for each non-word) and eye-movement measures (mean fixation duration and mean number of fixations per syllable) were analyzed using mixed-effects models with the lme4 (Bates et al., 2015) and lmerTest (Kuznetsova et al., 2017) packages.

For spelling accuracy, binary data were modeled with generalized linear mixed models (GLMMs) using a binomial distribution and logit link function. The first model included data from learning phases 1 to 3 to analyze encoding and learning progression. Fixed effects included group (two levels: good vs. poor spellers; good spellers as the reference level), learning phases (three levels: dictations after each of the three learning phases; first learning phase as the reference level), phoneme (two levels: non-words with the phoneme /ã/ vs. non-words with the phoneme /$\tilde{ɛ}$/; phoneme /ã/ as the reference level), and their interactions. Random intercepts were included for participants and items (non-words). Simple slope analyses were conducted using the emtrends function from the emmeans package (Lenth & Piaskowski, 2025). The full model was the following:

glmer(Accuracy score ~ Group × Learning cycle (or Testing phase) × Phoneme + (1|Participant) + (1|Non-word))(1)

A second GLMM included data from learning phase 3 and the delayed dictation, corresponding to the fourth testing phase, to analyze consolidation and retention. Because the model failed to converge with the same structure as Equation (1), we simplified the model and removed the phoneme factor. Akaike’s Information Criterion (AIC) indicated that this structure was the best-fitting model. Fixed effects of this model included group (two levels: good vs. poor spellers; good spellers as the reference level), testing phases (two levels: dictation after learning phase 3 and delayed dictation; third learning phase as the reference level), and their interaction. Random intercepts were included for participants and items. Again, simple slope analyses were conducted using the emtrends function from the emmeans package.

For the delayed recognition task, the models including group and phoneme, and even group alone, indicated a nearly singular fit. We then removed the random effects and conducted a logistic regression to analyze the recognition performance of children. Group (good vs. poor spellers) and phoneme (/ã/ vs. /$\tilde{ɛ}$/) were introduced as predictors of accuracy score, as well as their interaction. The model was fitted using a binomial distribution with a logit link function in R (R Core Team, 2023).

For the analysis of spelling error types, the distribution of error categories across learning cycles and the delayed dictation was examined. Given the zero-inflated nature of the data, the number of occurrences of each error type was averaged across dictations. Between-group and within-group comparisons were then carried out using independent-samples *t*-tests and paired *t*-tests, respectively.

For eye-movement measures, mean fixation duration and mean number of fixations per syllable were modeled with linear mixed-effects models (LMMs). Three categorical predictors were included as fixed effects: group (good vs. poor spellers; good spellers as the reference level), syllable type (inconsistent vs. consistent; inconsistent syllable as the reference level), and learning phase (three levels, with the first learning phase as the reference level). Random intercepts were included for participants and items as well. A series of models were compared using Akaike’s Information Criterion (AIC) to identify the best-fitting model. For both dependent variables, the optimal model included an interaction between group and syllable type, and a main effect of learning phase:

lmer(DV ~ Group × Syllable + Learning phase + (1|Participant) + (1|Non-word))(2)

The inclusion of learning phase as a main effect improved model fit compared to the model containing only the group × syllable interaction (for mean number of fixations: *p* < 0.05; for mean fixation duration: *p* = 0.062). Models including higher-order interactions (e.g., triple interaction) did not improve model fit and had higher AIC values. Post hoc pairwise comparisons were conducted to examine interactions, using Welch’s *t*-tests and paired *t*-tests, respectively, for between-subject and within-subject comparisons. Effect sizes (Cohen’s *d*) were calculated with the effsize package (Torchiano, 2020).

Interactions for accuracy models were plotted using the effect function from the effects package (Fox, 2003; Fox & Weisberg, 2019). All plots were visualized using ggplot2 package (Wickham, 2016) in R. Summary tables reporting significance levels, effect sizes, and AIC values were generated using the tab_model function from the sjPlot package (Lüdecke, 2024), and are provided in Appendix A.

## 3. Results

### 3.1. Behavioral Results

#### 3.1.1. Spelling Accuracy Results

**Figure 2:**
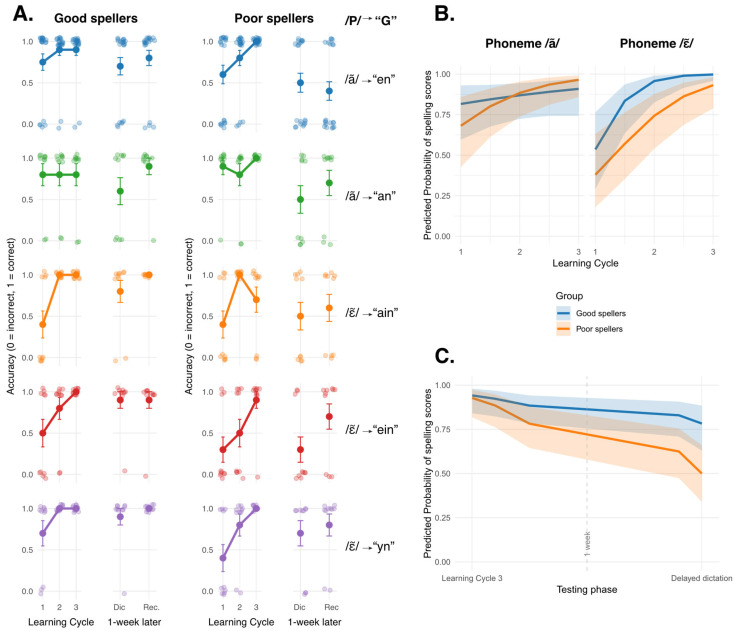
(**A**) Accuracy scores across behavioral measures for each non-word, by group. Dictation scores for the three learning cycles (1, 2, 3) are provided on the left of each panel, while scores for the one-week delayed dictation (Dic) and recognition task (Rec) appear on the right. Target phonemes (/P/) are presented between slashes, and graphemes (“G”) are shown in quotation marks. (**B**) Predicted probability of correct spellings are plotted as a function of learning cycles and target phoneme (/ã/ or /$\tilde{ɛ}$/), separately for good (blue) and poor spellers (orange). This figure illustrates initial encoding and learning progression. (**C**) Predicted probability of correct spellings are plotted as a function of testing phases (third learning phase and one-week delayed dictation), separately for good (blue) and poor spellers (orange). This figure illustrates consolidation and retention after a one-week delay.

As a first step, we used GLMM to examine group differences in spelling accuracy across the three learning phases and as a function of the target phoneme (/ã/ vs. /$\tilde{ɛ}$/). This model informed us about initial encoding and learning progression. Accuracy results for each P-G correspondence are presented in Figure 2A. Overall, learning progression differed between non-words containing the phoneme /ã/ and those with the phoneme /$\tilde{ɛ}$/ (*β* = −3.90, *SE* = 1.30, *z* = −3.00, *p* = 0.003), as shown in Figure 2B. Spelling accuracy also increased across learning cycles, but this effect was modulated by a significant Learning phase × Phoneme interaction (*β* = 2.56, *SE* = 0.87, *t* = 2.94, *p* = 0.003) and a significant triple interaction (*β* = −2.28, *SE* = 1.03, *t* = −2.21, *p* = 0.027), indicating the learning progression may differ between good and poor spellers for some non-words. See Table A1 in Appendix A.

To further examine these significant interactions, we conducted simple slope analyses using the emtrends function from the emmeans package in R (Lenth & Piaskowski, 2025). This analysis estimated the slope of learning phases separately for each group and each phoneme. All slopes of learning phases, except one, were different from zero, indicating accuracy scores increased significantly across learning phases (non-words with /ã/ for poor spellers: Estimate = 1.27, *SE* = 0.43, *z* ratio = 2.98, *p* = 0.003; non-words with /$\tilde{ɛ}$/ for good spellers: Estimate = 2.96, *SE* = 0.79, *z* ratio = 3.74, *p* < 0.001; and for poor spellers: Estimate = 1.55, *SE* = 0.38, *z* ratio = 4.07, *p* < 0.001). As illustrated in Figure 2B, good spellers obtained relatively high scores for non-words containing the phoneme /ã/ from the start and did not improve their performance a lot after the three learning phases. When comparing slopes between groups, no significant difference emerged (good vs. poor spellers for non-words with /ã/: Estimate = −0.87, *SE* = 0.56, *z* ratio = −1.54, *p* > 0.1; good vs. poor spellers for non-words with /$\tilde{ɛ}$/: Estimate = 1.41, *SE* = 0.87, *z* ratio = 1.62, *p* > 0.1).

We then used GLMM to analyze consolidation and retention curve over time. Only two testing phases were included in the model: the last learning phase (testing phase 3) and the testing phase corresponding to the delayed dictation (testing phase 4). The phoneme was not included in the analysis as the model failed to converge. As expected, children performed worse to the delayed dictation after one week (*β* = −1.50, *SE* = 0.60, *z* = −2.49, *p* = 0.012), reflecting partial forgetting over time (see Figure 2A,C). The interaction between the testing phase and the group was not significant. See Table A2 in Appendix A. Simple slope analyses confirmed that accuracy scores were lower in the delayed dictation, in comparison to the third learning phase’s dictation (good spellers: Estimate = −1.50, *SE* = 0.61, *z* ratio = −2.49, *p* = 0.013; poor spellers: Estimate = 2.55, *SE* = 0.56, *z* ratio = −4.54, *p* < 0.001). However, slopes did not differ between groups (Estimate = 1.05, *SE* = 0.82, *z* ratio = 1.28, *p* = 0.2), even if Figure 2C showed a steeper forgetting slope for poor spellers. They also tended to perform worse than good spellers in the delayed dictation.

The final stage of analysis was to verify the effect of the group and the phoneme on the delayed recognition task score. As GLMM indicated a nearly singular fit, we simplified the model structure and conducted a logistic regression. As predicted, a significant effect of group was found (OR = 0.20, 95% CI [0.06, 0.63], *p* = 0.008). Poor spellers scored significantly lower than good spellers on the recognition task. However, no effect was observed for either the phoneme (OR = 5.80, 95% CI [0.86, 115.10], *p* = 0.119) or the Group × Phoneme interaction (OR = 0.40, 95% CI [0.02, 3.67], *p* = 0.467). See Table A3 in Appendix A.

#### 3.1.2. Results of Spelling Error Analysis

**Figure 3:**
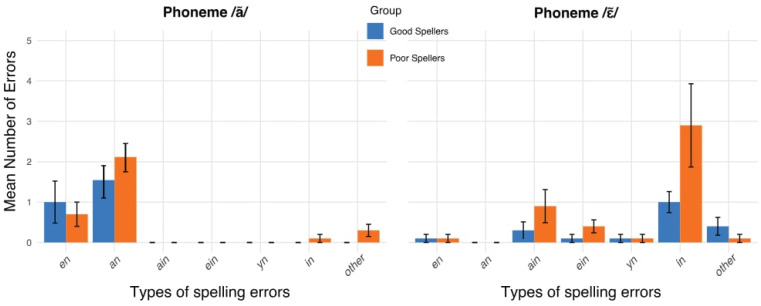
Mean number of spelling errors per target phoneme, averaged across all dictations (learning cycles and delayed dictation), are shown separately for good (blue) and poor spellers (orange). The x-axis displays the erroneous grapheme produced by children when spelling errors occurred. For instance, the use of the grapheme “en” instead of “an” occurred on average once in good spellers and less than once in poor spellers. Error bars represent ±1 SEM.

We also examined the types of spelling errors produced by participants and whether the two groups differed in their error patterns. Figure 3 presents the mean number of spelling errors per group as a function of error type for each phoneme, averaged across all dictations (learning cycles and delayed dictation). For the phoneme /ã/, the most frequent error involved confusion between the two alternative spellings—substituting “en” for “an” and vice versa. The mean frequency of these two spellings (“en”: M_good_ (SD) = 1.0 (1.63), M_poor_ (SD) = 0.7 (0.95); and “an”: M_good_ (SD) = 1.5 (1.27), M_poor_ (SD) = 2.1 (1.10)) did not differ significantly between groups (*p* > 0.3).

For the phoneme /$\tilde{ɛ}$/, which had three possible spelling alternatives in our study, errors reflected confusion among these options. Fewer errors involved the least frequent spelling “yn” in French (M_good+poor_ (SD) = 0.1 (0.32)), while an interesting pattern emerged with the intrusion of the “in” spelling, which is the most regular and frequent spelling for the phoneme /$\tilde{ɛ}$/ in French (Catach, 2014; Peereman et al., 2007). The grapheme “in” was by far the most frequent error used to spell the phoneme /$\tilde{ɛ}$/, particularly among poor spellers (M_good_ (SD) = 1.0 (0.82), M_poor_ (SD) = 2.9 (3.25)). However, there was no between group differences on the mean count of the occurrence of “in” error (*t*(18) = −1.79, *p* = 0.89). The frequency of errors involving “ain” and “ein” fell between that of “in” and “yn” (“ain”: M_good_ (SD) = 0.3 (0.67), M_poor_ (SD) = 0.9 (1.29); “ein”: M_good_ (SD) = 0.1 (0.32), M_poor_ (SD) = 0.4 (0.52)). Although the paired comparison between the mean frequency of the spelling errors “in” and “ain” was not significant in either group (*t*(9) < 1.6, *p* > 0.1), the mean frequency of “in” errors was significantly higher than that of “ein” (*t*(9) = 2.28, *p* = 0.049, Cohen’s *d* = 0.72) and “yn” (*t*(9) = 2.75, *p* < 0.03, Cohen’s *d* = 0.91) in the poor spellers group. A similar pattern was observed among good spellers, for whom the “in” spelling error also occurred more frequently than “ein” (*t*(9) = 3.25, *p* < 0.01, Cohen’s *d* = 1.03) and “yn” (*t*(9) = 2.86, *p* < 0.02, Cohen’s *d* = 0.91). These results showed that “in” was preferred over “ein” and “yn,” but not necessary over “ain”.

### 3.2. Eye-Tracking Results

#### 3.2.1. Mean Fixation Duration per Syllable

**Figure 4:**
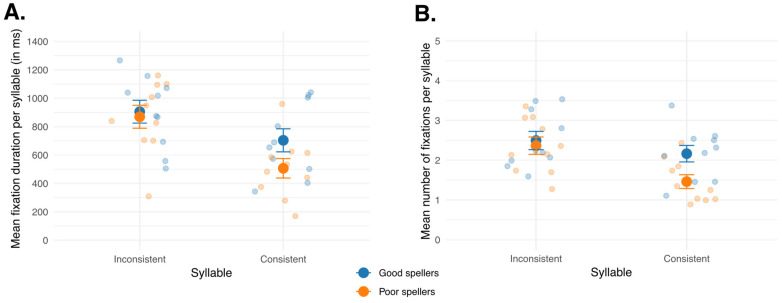
Illustrations of the interaction between the syllable type (inconsistent and consistent) and the group (good spellers in blue and poor spellers in orange) for the mean fixation duration per syllable (**A**) and the mean number of fixations per syllable (**B**). Mean fixation duration are provided in milliseconds (ms). Error bars represent ±1 SEM.

**Figure A1:**
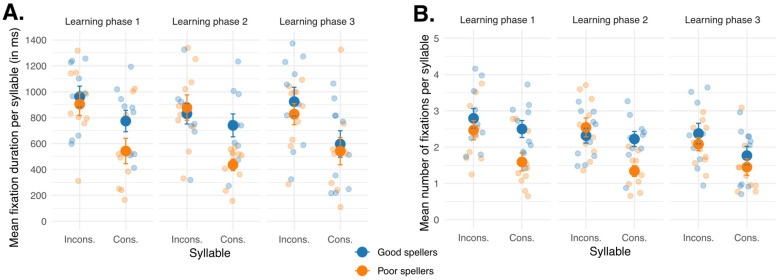
Mean fixation duration per syllable (**A**) and mean number of fixations per syllable (**B**) across the three learning phases, as a function of the syllable (inconsistent—incons. and consistent—cons.), and separately for good (blue) and poor spellers (orange). This figure illustrates the decrease in mean fixation duration and mean number of fixations per syllable across learning phases, as indicated in Table A4 and Table A5. Mean fixation duration are provided in milliseconds (ms).

Table A4, in Appendix A, reports the results of the LMM predicting mean fixation duration per syllable. There was no significant effect of group (*β* = −35.80, *SE* = 100.92, *t* = −0.36, *p* = 0.723). In contrast, mean fixation durations were significantly shorter for consistent than for inconsistent syllables (*β* = −201.66, *SE* = 41.69, *t* = −4.84, *p* < 0.001). A significant effect of learning phase also emerged, with mean fixation durations decreasing from the first to the second (*β* = −74.44, *SE* = 36.10, *t* = −2.06, *p* = 0.040) and from the first to the third learning phase (*β* = −73.47, *SE* = 36.10, *t* = −2.04, *p* = 0.042). This effect of learning phase is illustrated on Figure A1A, in Appendix A. Importantly, these effects were modulated by a significant Group × Syllable interaction (*β* = −161.29, *SE* = 58.96, *t* = −2.74, *p* = 0.006), indicating that the influence of syllable consistency differed between good and poor spellers (Figure 4A).

To further examine the significant Group × Syllable interaction, post hoc pairwise comparisons were conducted to compare mean fixation durations between groups for each syllable type (between-subject analyses) and between syllables within each group (within-subject analyses).

In the between-subject comparisons, no significant difference was found between good and poor spellers for the inconsistent syllable (Welch’s *t* (18) = 0.31, *p* = 0.757), indicating similar mean fixation durations across groups. A comparable pattern also emerged for the consistent syllable (Welch’s *t* (17.51) = 1.85, *p* = 0.081; Cohen’s *d* = 0.83), though the near significant result and the large effect size suggested a trend for good spellers to fixate longer on that syllable than poor spellers. The small sample size may explain the lack of significant statistical difference despite a large effect size.

In the within-subject analyses, for both groups, children fixated longer on the inconsistent syllable than on the consistent one (good spellers: paired *t*(9) = 2.78, *p* = 0.021; Cohen’s *d* = 0.79; poor spellers: paired *t*(9) = 7.22, *p* < 0.001; Cohen’s *d* = 1.50). However, the effect size was substantially larger for poor spellers, indicating that their attention was more strongly drawn to the inconsistent syllable.

#### 3.2.2. Mean Number of Fixations per Syllable

Table A5, in Appendix A, summarizes the LMM for the mean number of fixations per syllable. As with mean fixation duration, there was no significant main effect of group (*β* = −0.13, *SE* = 0.27, *t* = −0.48, *p* = 0.638). However, significant effects emerged for syllable and learning phase. The mean number of fixations decreased significantly across learning phases—from the first to the second (*β* = −0.23, *SE* = 0.09, *t* = −2.46, *p* = 0.014) and from the first to the third phase (*β* = −0.42, *SE* = 0.09, *t* = −4.46, *p* < 0.001). This effect is illustrated on Figure A1B, in Appendix A. In addition, participants made fewer fixations on consistent than on inconsistent syllables (*β* = −0.33, *SE* = 0.11, *t* = −3.04, *p* = 0.002). Importantly, the Group × Syllable interaction was significant (*β* = −0.57, *SE* = 0.15, *t* = −3.72, *p* < 0.001) indicating that the effect of syllable consistency differed between good and poor spellers (see Figure 4B).

To further examine this interaction, post hoc pairwise comparisons were conducted. Between-subject comparisons revealed no significant difference between good and poor spellers for the inconsistent syllable (Welch’s *t*(17.96) = 0.41, *p* = 0.686), indicating comparable fixation patterns. However, for the consistent syllable, a significant difference emerged: good spellers made more fixations than poor spellers (Welch’s *t*(17.46) = 2.60, *p* = 0.018; Cohen’s *d* = 1.16).

Within-subject comparisons showed slightly different patterns than those for mean fixation durations. For good spellers, the number of fixations did not differ significantly between consistent and inconsistent syllables (paired *t*(9) = 1.82, *p* = 0.103), indicating comparable processing of both syllable types. In contrast, poor speller showed a clear effect of syllable, with significantly fewer fixations on the consistent syllable compared to the inconsistent one (paired *t*(9) = 8.18, *p* < 0.001; Cohen’s *d* = 1.34).

## 4. Discussion

The present study examined how children learn the spelling of inconsistent non-words. Dictation scores and eye-tracking measures were combined to better understand learning progression across exposures, and long-term retention was assessed one week later through delayed dictation and recognition tasks.

Children with good and poor spelling skills were included in the study and classified according to their performance on an irregular word dictation task from a standardized assessment tool (BALE; Jacquier-Roux et al., 2010). Thus, spelling performance was used as a proxy to examine differences in orthographic learning mechanisms. The first objective was to analyze learning progression by linking spelling accuracy and eye-movement measures. The second objective was to examine the influence of the inconsistent syllable on the visual exploration of non-words during learning phases. Each objective will now be discussed in light of our hypotheses and results obtained.

### 4.1. Orthographic Learning Progression Reflected in Spelling Accuracy, Spelling Errors, and Eye Movements

#### 4.1.1. Orthographic Learning Progression Based on Spelling Accuracy

As expected, both groups improved their dictation scores between the first and the third learning cycles, indicating that all children were able to encode and refine orthographic representations after only a few exposures. Importantly, no group difference emerged in learning progression across cycles, suggesting comparable learning trajectories in good and poor spellers. This pattern is consistent with previous findings showing that children with spelling difficulties can achieve performance levels comparable to their peers with repeated practices (Binamé et al., 2015; Mehlhase et al., 2019). However, the present design extends these findings by explicitly modeling learning trajectories across cycles, thereby allowing a clearer distinction between acquisition and later consolidation processes.

Furthermore, our results showed that one week later, a significant decrease in performance was observed between the final learning cycle and the one-week delayed dictation. Both groups exhibited lower scores after the delay, reflecting the natural course of memory decay (see Radvansky et al., 2022). Nevertheless, as predicted, poor spellers tended to show difficulty in accurately spelling the learned non-words in the delayed dictation, and performed significantly worse than good spellers in the delayed recognition task. Typically, recognition tasks yield higher accuracy than dictation because they rely on partial activation of orthographic representations rather than full recall (Bosman & Van Orden, 1997; Perfetti, 1997; Wang et al., 2011). Visual inspection of accuracy scores in both tasks supported this tendency. Because good spellers obtained higher scores in the delayed recognition task, this suggests that their orthographic representations of non-words were more precise and more robust than those of poor spellers, even if both groups showed similar performances in the delayed dictation, perhaps a little higher for good spellers.

The recognition task used in this study may have been particularly demanding for poor spellers. Each item presented four phonologically plausible alternatives, with errors on the inconsistent P-G correspondence and in orthographic details such as silent letters or consonant doubling (see Procedure section for examples). Because all alternatives are plausible in French, accurate discrimination required well-specified orthographic representations. This pattern therefore suggests that poor spellers’ difficulties primarily reflect impairments in consolidation and retrieval processes rather than limitations in initial encoding. This interpretation is in line with Binamé et al. (2015), who showed that children with DD could learn the spelling of pseudo-words after repeated practice, but failed to maintain these gains after one week. The authors attributed this drop to deficits in the consolidation and retrieval of newly learned spellings. Similar findings were also reported by Mehlhase et al. (2019) who observed that children with spelling difficulties, whether or not with associated reading difficulties, were unable to retain newly learned pseudo-word spellings after only two hours, despite showing learning during the training session.

Together, these offline results indicate that orthographic learning difficulties in poor spellers may arise from impaired consolidation processes (Binamé et al., 2015; Mehlhase et al., 2019) that hinder the stabilization and long-term maintenance of newly learned orthographic representations (Eustache et al., 2016; Guillery-Girard et al., 2008). 

#### 4.1.2. Impact of Phonemes-to-Graphemes Correspondences’ Characteristics on Orthographic Learning and Spelling Errors

When analyzing the learning curves as a function of the target phoneme, good spellers did not improve their spelling scores in the non-words with the phoneme /ã/. Their performance plateaued, whereas poor spellers improved across the learning phases. Nevertheless, learning progressions were similar between groups. For non-words with the phoneme /$\tilde{ɛ}$/, both groups significantly improved their accuracy scores and did not differ from each other in their learning progression. We then examined the spelling errors to better understand those differences between target phonemes and discussed the impact of P-G correspondences’ characteristics (i.e., frequency and regularity) on errors.

For the phoneme /ã/, only two graphemes (“en” and “an”) can represent the sound, and both are highly frequent in French (Catach, 2014; Peereman et al., 2007; Ruberto et al., 2025). They provide few statistical cues to guide spelling, making memorization uncertain for all participants (Ruberto et al., 2025) and leading to spelling errors (Daigle et al., 2020). Qualitative analyses of spelling errors point in that direction: both good and poor spellers tended to alternate between “en” and “an” when uncertain. Indeed, children had approximately a 50% chance of using the correct grapheme to spell the non-words, though this probability was likely slightly higher for the grapheme “en”, given its greater occurrence (two instances) compared to the grapheme “an” (one instance) in the learning task. This may explain why good spellers showed a flatter slope. According to the IMP model, encoding and retrieval of the spelling of a word is harder when its orthographic representation does not rely on multiple convergent cues, such as high frequency or high regularity (Treiman, 2017). Moreover, exceptional errors were made by poor spellers, introducing more variability in their error types in comparison to good spellers. These errors were either phonological (e.g., “lufen” spelled “lufin”, with a final /$\tilde{ɛ}$/) or the addition of a silent letter (e.g., “lanti” spelled “lantie”). Those errors appeared rarely across learning cycles among poor spellers. Given their low number, they are difficult to interpret definitively but could be related to a phonological confusion between /ã/ and /$\tilde{ɛ}$/, to an interference with non-words with /$\tilde{ɛ}$/—which all ended with the target phoneme –, to an attentional error, or even to an influence of general orthographic knowledge (for silent letters).

In contrast, the phoneme /$\tilde{ɛ}$/ was spelled three ways in our study, and all these spellings were relatively infrequent in French, contrary to the grapheme “in” which is the most common orthographic representation of this sound (Catach, 2014; Peereman et al., 2007). The grapheme “in” was intentionally excluded from the stimuli to assess how children learn less common spellings. When examining errors, both groups mainly alternated between the most frequent grapheme “in” and the grapheme “ain”, the latter being one of the correct spellings in the task. In good spellers, errors involving the grapheme “in” were mostly concentrated at the beginning of the learning cycles, suggesting a progressive stabilization of orthographic representations. Furthermore, poor spellers persisted using the grapheme “in” despite repeated exposure to the other spelling alternatives used in the study. When the correct spelling had not yet been consolidated, both groups relied on the most probable grapheme in the French writing system, reflecting the use of their general orthographic knowledge (Pacton & Afonso-Jaco, 2015; Pacton et al., 2013; Treiman & Kessler, 2025). Poor spellers still used the grapheme “in” across learning cycles, indicating that their orthographic representations were less stable over time.

To further examine the mechanisms underlying these difficulties, the following section focuses on eye-tracking measures. By capturing how children allocate their attention and process written information in real time, these measures may reveal subtle aspects of the learning process that are not accessible through behavioral data alone. Therefore, they may help refine and nuance our interpretation of the present results, particularly regarding potential differences in encoding strategies between good and poor spellers.

#### 4.1.3. Contribution of Eye Movements to Orthographic Learning Progression

A main effect of the learning phase was observed in the LMM, indicating a decrease in both mean fixation durations and mean number of fixations per syllable across exposure. As the interaction between learning phase and group did not significantly improve the model, there was no evidence of group differences. Therefore, both good and poor spellers exhibited this overall reduction in eye-movement measures, consistent with our expectations and with findings reported in children (Joseph & Nation, 2018; van Viersen et al., 2022) and in adults (Ginestet et al., 2020b; Joseph et al., 2014). This decrease in mean number of fixations and mean fixation durations per syllable across exposures reflects more efficient visual processing. Indeed, shorter and fewer fixations are typically associated with higher word familiarity and frequency during reading (e.g., Joseph et al., 2013, 2014; Rayner, 2009; Schroeder et al., 2015). When applied to a learning context, this pattern has been interpreted as evidence that orthographic representations are progressively established in the orthographic lexicon, leading to faster visual processing with repeated encounters (Ginestet et al., 2020b; Joseph & Nation, 2018; van Viersen et al., 2022). Similar effects have also been reproduced computationally using the BRAID-Learn model, which simulates the evolution of eye movements patterns during the development of orthographic representations of words (Ginestet et al., 2022).

In the context of the present learning task, children were not required to read the stimuli aloud, as each non-word’s pronunciation was played through speakers. Consequently, eye movements did not reflect decoding or lexical access processes, but rather the visual encoding of the orthographic form while its pronunciation was already known. Previous research has shown that visual attention plays a crucial role in the acquisition of new orthographic representations (Bosse et al., 2015; Ginestet et al., 2020b), especially when auditory information is simultaneously available (e.g., Nation et al., 2007). One could have expected poor spellers to show a smaller reduction in fixation duration or a slower improvement in eye-movement efficiency if their visual-orthographic encoding was impaired. While no difference was found between groups on global eye-movement measures across learning phases, a striking difference emerged when examining how eye movements were distributed across inconsistent and consistent syllables, as will be discussed later.

Linking eye-tracking measures and dictation scores across learning cycles provided complementary insights into the learning process in good and poor spellers. This approach made it possible to track learning progression step by step and directly relate behavioral performance to visual processing dynamics. To our knowledge, this study is the first to do so, as previous works (see Ginestet et al., 2020b; Joseph & Nation, 2018; van Viersen et al., 2022) typically assessed learning outcomes only after all exposure phases. The current findings reinforce the interpretation that reduced fixation durations and fixation counts correspond to the gradual development of orthographic representations, given their parallel increase in dictation scores across learning cycles.

### 4.2. Influence of Inconsistencies on Visual Exploration Patterns During Encoding

In contrast to our predictions, a difference between groups in visual exploration patterns emerged when examining the distribution of eye-movement measures across the two syllables of the non-words. The present results showed that good spellers looked longer at the inconsistent syllable but did not fixate on it more often than on the consistent syllable. Poor spellers, however, fixated more often and for longer durations on the inconsistent syllable, and this effect was even larger than in good spellers. Importantly, poor spellers allocated less attention to the consistent syllable compared to good spellers. These findings suggest that poor spellers adopt an imbalanced attentional allocation pattern, focusing disproportionately on the inconsistent syllable at the expense of the rest of the word (i.e., the consistent syllable). This imbalanced processing may have consequences for encoding: successfully memorizing an inconsistent spelling requires forming an integrated word-specific orthographic representation that binds the particular grapheme to the complete word. If children focus exclusively on the inconsistency in isolation, they may struggle to anchor it to a specific lexical entry, thereby compromising the formation of a unified and stable orthographic representation. Therefore, the visual encoding of the spelling of non-words may differ between groups. Poor spellers appear to adopt a more analytical processing pattern, focusing primarily on the inconsistent syllable.

This interpretation remains partly inferential, as we cannot directly measure the “analytic” or “holistic” nature of encoding from eye-tracking data alone. However, this repeated emphasis on the inconsistent syllable combined with reduced attention to the rest of the word provides empirical evidence that poor spellers process words more fragmentarily than good spellers. An alternative and complementary interpretation is that poor spellers are sensitive to orthographic inconsistencies but require more time and/or exposures to successfully encode and consolidate these unpredictable segments into long-term memory. These two interpretations are not mutually exclusive: initial fragmentary encoding (reflected by imbalanced attentional allocation) could make subsequent consolidation more difficult, thereby necessitating more time to stabilize orthographic representations and contributing to the weaker delayed retention observed in poor spellers (Binamé et al., 2015; Mehlhase et al., 2019).

In contrast, good spellers looked more often at the consistent syllable and tended to fixate on it longer than poor spellers, reflecting a more balanced allocation of attention across the entire word. This more uniform distribution of attention may have facilitated the formation of more unified orthographic representations, allowing them to establish stronger connections between the inconsistent syllable and the overall orthographic form. Good spellers seemed to adopt a more global processing pattern, supporting the formation of complete and durable orthographic representations, which may explain their better long-term retention. This interpretation aligns with Bosse et al. (2015), who showed that long-term orthographic learning is more effective when words are presented and encoded as whole units rather than fragmented parts.

This finding can be further interpreted through the BRAID-Learn model, which posits that visual attention is dynamically deployed toward elements that are more difficult to process—here, the inconsistent syllable—to optimize encoding (Ginestet et al., 2022). From this perspective, the increased fixation duration and number of fixations on inconsistent syllables may reflect adaptive visual-attentional mechanisms recruited to support encoding of more complex segments. However, when this attentional allocation becomes so imbalanced that it neglects certain letters of a word, it may compromise the formation of integrated orthographic representations. Accuracy scores suggest that, despite the increased attention allocated to the inconsistent syllable during encoding, poor spellers struggled with long-term retention of the learned forms. This pattern suggests that difficulties may stem from both initial fragmentary encoding and subsequent consolidation deficits.

When related to spelling errors, these findings suggest that poor spellers may experience difficulties during the encoding and/or consolidation processes, as they persisted in using the grapheme “in” even though it was absent from non-words with /$\tilde{ɛ}$/. Because consolidation depends on the precision and robustness of the initial encoding (Fayol & Jaffré, 2024), poorly specified orthographic representations are unlikely to become fully stabilized in long-term memory, resulting in weaker retention (Binamé et al., 2015). Nevertheless, this interpretation does not exclude the possibility that poor spellers may also face additional difficulties in consolidation and long-term retention, as suggested by the pronounced decline observed in the recognition task and the similar trend found in the delayed dictation after one week.

### 4.3. Extension of the Findings to Children with Dyslexia-Dysorthographia

Although this study did not directly compare children with and without DD, we believe that our findings can reasonably be extended to this population. First, the poor spellers group included five children (50%) with either a formal diagnosis or a substantiated diagnostic hypothesis of DD, established by qualified professionals (e.g., speech-language pathologists, neuropsychologists). Parents of these children also confirmed persistent spelling and reading difficulties in their child, which were observed with our own comprehensive assessments (including word dictations and word reading tasks). The remaining five children, although not suspected of DD, exhibited profiles characterized by major and selective difficulties with irregular word spelling. They mostly scored below average when compared to the spelling assessment’s normative data, which was used for descriptive purposes only. Children exhibited slower reading fluency and lower reading accuracy, while showing relatively preserved performance on non-word spelling. See Table 1 for descriptive data. Selective difficulties with irregular or inconsistent orthographic patterns alongside preserved phonological spelling abilities are consistent with profile of some children with DD (Carroll et al., 2025; Daigle et al., 2016; Stanké, 2016).

Second, our findings closely align with prior research documenting consolidation deficits in children with confirmed DD diagnoses: these children could obtain similar performance levels to their peers after repeated practice but failed to maintain what they had learned over time (Binamé et al., 2015; Mehlhase et al., 2019). Indeed, poor spellers in the present study showed comparable initial learning trajectories but exhibited marked difficulties at the one-week delayed tasks. The eye-tracking data further revealed disproportionate attention to the inconsistent syllable at the expense of the other syllable, suggesting encoding strategies that may compromise the quality and long-term stability of orthographic representations.

Given the substantial proportion of children with confirmed or suspected DD in the sample and the strong convergence between our findings and previous research on consolidation and retrieval deficits in children with DD, we believe that our results, particularly those concerning the consolidation and retrieval of newly learned orthographic representations for inconsistent words, can reasonably be extended to this population. Nevertheless, future studies involving larger samples of children with formally diagnosed DD will be necessary to further confirm and refine these interpretations.

### 4.4. Practical Implications

The findings of the present study have direct implications for educational and clinical practices aimed at supporting children who experience difficulties with French spelling, particularly when learning words containing inconsistent P-G correspondences. Our results indicate that poor spellers show specific weaknesses in consolidating and retrieving newly learned orthographic representations, even when their initial encoding performance appears comparable to that of good spellers. This pattern suggests that spelling difficulties may remain undetected if assessment focuses exclusively on immediate accuracy. Evaluating spelling performance after a delay, by re-administering the same dictation one or two weeks later, may therefore provide a more sensitive means of identifying children who struggle with orthographic retention and may guide the selection of appropriate instructional or therapeutic strategies.

The persistence of spelling errors across learning cycles, combined with the significant decline in performance after a one-week delay, underscores that inconsistent spellings cannot be left to incidental exposure and require explicit, systematic instruction. Our findings revealed a significant triple interaction between group, learning cycle, and phoneme, indicating that the challenges posed by inconsistent P-G correspondences may vary as a function of their statistical properties in the language (e.g., frequency, regularity). Notably, despite repeated exposure to less frequent spellings (“ain”, “ein”, “yn”), poor spellers persistently substituted target graphemes by the grapheme “in”, the most frequent one in French for the phoneme /$\tilde{ɛ}$/. This intrusion of high-frequency competitor grapheme suggests that instructional efforts should prioritize low-frequency or weakly regular spellings, as these are more vulnerable to interference from statistical regularities. Explicitly, contrasting the target grapheme with more frequent alternatives (e.g., “This word uses ‘ain’ to spell the sound /$\tilde{ɛ}$/, not the usual ‘in’.”) may help learners inhibit default responses and strengthen the encoding of exceptional spellings. These findings also indicate that simply repeating the same instructional procedure across learning cycles may be insufficient. Teaching methods may need to incorporate retrieval practice, spaced review, or explicit comparison with competitor graphemes to better support consolidation, particularly for poor spellers. Moreover, the fragility of retention observed in poor spellers one week after learning underscores the importance of spaced practice schedules (e.g., revisiting words after one day, one week, and two weeks) rather than massed repetition, in line with recent evidence on interleaved and distributed practice (Klimovich & Richter, 2025).

The eye-tracking results provide further insight into how attentional allocation during encoding may contribute to consolidation difficulties. Poor spellers allocated disproportionate attention to inconsistent syllables while devoting significantly less attention to consistent syllables, resulting in an unbalanced processing strategy focused primarily on the orthographic difficulty. In contrast, good spellers distributed their attention more evenly across the entire word, including both consistent and inconsistent syllables. This more balanced allocation may have facilitated the formation of integrated orthographic representations that bind the distinctive graphemic feature to the complete word form, rather than encoding it as an isolated element. This interpretation aligns with evidence showing that orthographic learning is more effective when words are encoded as wholes rather than as one syllable at the time (Bosse et al., 2015; Chaves et al., 2020). From a clinical and instructional perspective, these findings suggest that interventions should explicitly guide poor spellers toward more balanced visual processing strategies. For instance, training exercises could encourage learners to attend to the entire word before focusing on its orthographic particularities, or to use whole-word rehearsal strategies that integrate the distinctive spelling within the orthographic form. Connecting orthographic specificities to semantic or morphological features may also promote more unified encoding. Such approaches are exemplified in instructional materials like *Mon orthographe illustrée* (Valdois et al., 2017b), which uses visual-semantic associations to anchor spelling patterns within meaningful representations. For example, associating the doubled consonant in the French word “*tunnel*” (tunnel) with the two openings of a tunnel embeds the orthographic detail (here, the doubled consonant) within the word’s meaning, therefore supporting both retention and retrieval. Providing immediate and detailed feedback during learning cycles, by highlighting both correct elements and errors across the entire word, may further reinforce integrated representations. Such meaning-based, whole-word, and feedback approaches may also increase engagement during learning, which could be particularly beneficial for less proficient spellers.

### 4.5. Limitations and Future Research

This study is among the first to examine the impact of inconsistencies on orthographic learning using eye-tracking, allowing visual processing during multiple exposures to be directly linked to spelling performance across learning cycles. Previous eye-tracking research on orthographic learning has primarily focused on typically developing readers and spellers. By comparing good and poor spellers, the present study provides novel insights into orthographic learning mechanisms in children with spelling difficulties. The results obtained for the poor spellers group, which included five children with DD, may offer preliminary indications regarding orthographic learning processes in individuals with DD.

Despite these contributions, several methodological limitations must be acknowledged. First, the relatively small number of non-words to learn (six in total) may have limited both statistical power and ecological validity. Future research should include a larger and more diverse set of stimuli with varying syllabic structures (e.g., monosyllabic and trisyllabic words), which would better reflect the range of orthographic patterns encountered in natural reading and spelling contexts. This would also provide more robust data for modeling learning trajectories. A follow-up study using trisyllabic non-words is currently underway and will extend the present findings to more complex orthographic structures. 

Second, the graphemes used to spell inconsistencies differed in frequency and regularity. This choice was intentional in the original assessment tool, as it reflects two major types of inconsistencies in the French writing system: spellings that are frequent and evenly distributed (i.e., “en” and “an”), and others that are less frequent and more asymmetrical (i.e., “ain”, “ein” and “yn”). While this design allowed us to discuss how children learn orthographic forms that vary in graphemic distribution, it also limited experimental control over grapheme length, number of competing spellings, and frequency. Future research could attempt to isolate these factors more precisely, for instance by comparing graphemes that are structurally similar but differ in frequency, or by manipulating frequency independently of grapheme regularity.

Third, the sample size was small (ten participants per group), which may have reduced statistical power and limited the detection of subtle between-group differences. The high interindividual variability in eye-tracking measures further complicates the identification of group-level differences. In addition, because groups based on DD diagnosis were unbalanced, children were grouped according to their spelling skills. Although poor spellers did not all have a formal DD diagnosis, their spelling and reading profile was closer to the one of children with DD, permitting to extend our results to the population with DD. Replication with larger samples, including a greater number of children with a confirmed DD diagnosis, is therefore essential to confirm and strengthen the patterns observed in the present study. Future studies would also benefit from recruiting more diverse samples, including children from different socio-economic backgrounds, multilingual contexts, and those with comorbid developmental disorders (e.g., ADHD, developmental language disorder), as these factors may interact with orthographic learning mechanisms.

Beyond these methodological considerations, future research should further investigate the dynamic interplay between visual attention, explicit learning, and consolidation processes in orthographic learning. Longitudinal designs following the same children over extended periods would provide valuable insights into the stability of orthographic representations and the long-term retention of inconsistent spellings, and would address whether early consolidation difficulties persist or diminish with development and continued literacy exposure. Moreover, eye-tracking offers a particularly valuable tool for examining how attentional allocation during encoding relates to later consolidation outcomes. Combining eye-tracking with electrophysiological or neuroimaging measures could further elucidate the temporal dynamics of these processes.

Finally, intervention studies examining the effectiveness of specific instructional approaches, such as visuo-semantic strategies, spaced repetition protocols, or multisensory encoding methods, would directly complement the practical implications outlined in this study. Eye-tracking could also be used as an outcome measure in such interventions, allowing researchers to assess whether targeted instructional strategies, such as encouraging poor spellers to distribute visual attention more evenly across the entire word, successfully modify attentional patterns during learning.

## 5. Conclusions

To our knowledge, this study is the first to examine how children with good and poor spelling skills learn inconsistent spellings through repeated exposure, combining dictation scores and eye-tracking measures. Although both groups learned the spelling of non-words and showed a similar learning progression, they did not appear to process non-words in the same way. Poor spellers allocated more attention to inconsistent syllables, at the expense of consistent syllables, suggesting a more analytical and less integrated encoding process. After one week, their spelling accuracy declined, which suggests that the orthographic memory traces were less stable over time. Whether this reflects weaker encoding, reduced consolidation, or both remains to be clarified. Thus, when children made errors on inconsistencies, they often used P-G correspondences that are more frequent in their writing system, reflecting their reliance on general orthographic knowledge. Overall, our findings highlighted the interplay between encoding, consolidation, and retrieval of orthographic representations in memory, offering new perspectives for understanding orthographic learning difficulties. Although the present study did not directly compare children with and without dyslexia-dysorthographia, the findings may nevertheless be informative for this population.

## Figures and Tables

**Table 1:** Groups’ characteristics.

		Good Spellers	Poor Spellers	*t*-Value		Cohen’s *d*
	Age (months)	133.1 (11.18)	125.3 (12.58)	1.466		0.66
	School grade	3rd grade: 1	3rd grade: 4	---		---
		4th grade: 2	4th grade: 1	---		---
		5th grade: 4	5th grade: 4	---		---
		6th grade: 3	6th grade: 1	---		---
	Gender	4 M, 6 F	2 M, 8 F	---		---
Spelling skills	Regular words (/10)	9.6 (0.52)	8.1 (1.29)	3.421	**	1.53
(BALE)	Irregular words (/10)	9.5 (0.85)	4.4 (1.90)	7.757	***	3.47
	Non-words (/10)	9.8 (0.42)	9.4 (1.58)	0.775		0.35
Reading skills	Regular words (score) (/20)	19.6 (0.70)	17.6 (2.84)	2.165	*	0.97
(BALE)	Regular words (speed) (s)	16.5 (4.36)	28.6 (13.96)	−2.626	*	−1.17
	Irregular words (score) (/20)	17.6 (2.95)	11.1 (5.97)	3.086	**	1.38
	Irregular words (speed) (s)	18.8 (7.00)	34.5 (18.57)	−2.500	*	−1.12
	Non-words (score) (/20)	18.0 (2.87)	15.1 (3.90)	1.894	*	0.85
	Non-words (speed) (s)	27.5 (6.82)	36.8 (12.70)	−2.042	*	−0.91
Phonological	Initial phoneme deletion (score) (/10)	8.7 (2.06)	8.8 (1.14)	−0.135		−0.060
awareness (BALE)	Final phoneme deletion (score) (/10)	7.6 (2.72)	8.5 (1.43)	−0.927		−0.414
Working memory	Forward digit span	8.3 (1.64)	6.8 (1.03)	2.451	*	1.096
(CELF CDN-F)	Backward Digit span	5.2 (1.69)	4.4 (0.84)	1.342		0.600
Visual attention span	% of letters accurately reported	79.1 (18.87)	75.8 (9.85)	0.490		0.219
(Evadys)						

Note. Means and standard deviations (SD), where SD are provided in brackets (). Gender: M = male (boys), F = female (girls). Welch’s one-tailed *t*-tests results: *** *p* < 0.001; ** *p* < 0.01; * *p* < 0.05.

**Table 2:** Non-words list, frequency and regularity of P-G correspondences.

Phoneme	Non-Word	P-G Correspondence	Frequency	Regularity
/ã/	“lufen” /lyfã/ “tenvo” /tãvo/	/ã/ → “en”	40,584.17	49.34
	“lanti” /lãti/	/ã/ → “an”	35,513.72	43.17
/$\tilde{ɛ}$/	“notain” /nɔt$\tilde{ɛ}$/	/$\tilde{ɛ}$/ → “ain”	3733.79	16.75
	“ridein” /ʁid$\tilde{ɛ}$/	/$\tilde{ɛ}$/ → “ein”	655.12	2.94
	“mulyn” /myl$\tilde{ɛ}$/	/$\tilde{ɛ}$/ → “yn”	5.12	0.02

Note. The orthographic form of non-words is displayed in quotation marks (“ ”). The pronunciation with the International Phonetic Alphabet (IPA) is provided between slashes (/). Frequency and regularity were taken from the Manulex-Infra database (Peereman et al., 2007), based on a corpus of a million written words from children’s literature books. Frequency represents the total frequency of P-G correspondences by token in 3rd and 5th grades, and it is estimated per million written words. High frequency P-G correspondences exhibit higher numbers while low frequency P-G correspondences are closer to zero. Regularity represents the consistency of P-G correspondences (total consistency by token) in percentage, based on the P-G correspondence written frequency divided by the frequency of all P-G correspondences for a specific phoneme (e.g., written frequency of the P-G correspondence /$\tilde{ɛ}$/ → “ain” divided by written frequency of all P-G correspondences /$\tilde{ɛ}$/ → “in”, “ain”, “ein”, etc.), also in 3rd and 5th grades. Regular (or more consistent) P-G correspondences are closer to a hundred percent while inconsistent P-G correspondences are closer to zero.

## Data Availability

The data presented in this study are available upon request from the corresponding author. The data are not publicly available due to privacy or ethical restrictions.

## Abbreviations

The following abbreviations are used in this manuscript:

ADHD	Attention deficit hyperactivity disorder
AIC	Akaike’s Information Criterion
BALE	*Batterie Analytique du Langage Écrit* (Analytical test battery of written language)
BOSS	Bank of Standardized Stimuli
DD	Dyslexia-dysorthographia (stands for Dyslexia in French)
IMP model	Integration of Multiple Patterns model
GLMM	Generalized Linear Mixed Model
LMM	Linear Mixed-effects Model
P-G correspondences/mappings	Phonemes-to-graphemes correspondences/mappings

## Appendix A

**Table A1:** Results from the General Linear Mixed Models with spelling accuracy as binary dependent variable. Group, learning phase (1 to 3) and phoneme as fixed effects. Reference levels were “Good spellers”, “Learning phase 1” and “Phoneme /ã/”. Participants and non-words were included as random intercepts. The model was run on 360 observations. Statistical significance of predictors was assessed using likelihood ratio tests (*p*), and significant values are in bold.

Predictors	Odds Ratios	CI	*p*
Intercept	2.95	0.59–14.81	0.188
Group [poor]	0.20	0.02–1.89	0.161
Learning phase	1.50	0.73–3.08	0.268
Phoneme /$\tilde{ɛ}$/	0.02	0.00–0.26	**0.003**
Group [poor] × Learning phase	2.38	0.79–7.16	0.123
Group [poor] × Phoneme /$\tilde{ɛ}$/	10.69	0.43–262.61	0.147
Learning phase × Phoneme /$\tilde{ɛ}$/	12.89	2.35–70.76	**0.003**
Group [poor] × Learning phase × Phoneme /$\tilde{ɛ}$/	0.10	0.01–0.78	**0.027**
Random Effects			
σ^2^	3.29		
τ_00 (Participant)_	0.93		
τ_00 (Non-word)_	0.09		
ICC	0.24		
N_Participant_	20		
N_Non-word_	6		
Observations	360		
Marginal R^2^/Conditional R^2^	0.385/0.530		

Note. AIC: 315.4.

**Table A2:** Results from the General Linear Mixed Models with spelling accuracy as binary dependent variable. Group and testing phase (3 and 4, the fourth corresponding to the delayed dictation) as fixed effects. Reference levels were “Good spellers” and “Testing phase 3”. Participants and non-words were included as random intercepts. The model was run on 240 observations. Statistical significance of predictors was assessed using likelihood ratio tests (*p*), and significant values are in bold.

Predictors	Odds Ratios	CI	*p*
Intercept	1472.34	16.44–131,887.26	**0.001**
Group [poor]	18.39	0.04–7807.07	0.346
Testing phase	0.22	0.07–0.73	**0.013**
Group [poor] × Testing phase	0.35	0.07–1.74	0.200
Random Effects			
σ^2^	3.29		
τ_00 (Participant)_	0.30		
τ_00 (Non-word)_	0.07		
ICC	0.10		
N_Participant_	20		
N_Non-word_	6		
Observations	240		
Marginal R^2^/Conditional R^2^	0.254/0.330		

Note. AIC: 222.4.

**Table A3:** Results from the logistic regression model with spelling accuracy as binary dependent variable. Group and phoneme were included as predictors. Reference levels were “Good spellers” and “Phoneme /ã/”. The model was run on 120 observations. Statistical significance of predictors was assessed using likelihood ratio tests (*p*), and significant values are shown in bold.

Predictors	Odds Ratios	CI	*p*
Intercept	5.00	2.08–14.81	**0.001**
Group [poor]	0.20	0.06–0.63	**0.008**
Phoneme /$\tilde{ɛ}$/	5.80	0.86–115.10	0.119
Group [poor] × Phoneme /$\tilde{ɛ}$/	0.40	0.02–3.67	0.467
Observations	120		
R^2^ Tjur	0.159		

**Table A4:** Results from the Linear Mixed Models with the mean fixation duration per syllable (ms) as dependent variable. Group, syllable and learning phase as fixed effects. Reference levels were “Good spellers”, “Inconsistent syllable” and “Learning phase 1”. Participants and non-words were included as random intercepts. The model was run on 720 observations. Statistical significance of predictors was assessed using likelihood ratio tests (*p*), and significant values are in bold.

Predictors	Estimates	CI	*p*
Intercept	954.76	797.29–1112.22	**<0.001**
Group [poor]	−35.80	−233.94–162.34	0.723
Syllable [consistent]	−201.66	−283.51–−119.81	**<0.001**
Learning phase 2	−74.44	−145.32–−3.55	**0.040**
Learning phase 3	−73.47	−144.35–−2.58	**0.042**
Group [poor] × Syllable [consistent]	−161.29	−277.04–−45.54	**0.006**
Random Effects			
σ^2^	156,426.36		
τ_00 (Participant)_	42,235.91		
τ_00 (Non-word)_	5432.33		
ICC	0.23		
N_Participant_	20		
N_Non-word_	6		
Observations	720		
Marginal R^2^/Conditional R^2^	0.114/0.321		

Note. AIC: 10,728.7.

**Table A5:** Results from the Linear Mixed Models with the mean number of fixations per syllable (ms) as dependent variable. Group, syllable and learning phase as fixed effects. Reference levels were “Good spellers”, “Inconsistent syllable” and “Learning phase 1”. Participants and non-words were included as random intercepts. The model was run on 720 observations. Statistical significance of predictors was assessed using likelihood ratio tests (*p*), and significant values are in bold.

Predictors	Estimates	CI	*p*
Intercept	2.71	2.29–3.14	**<0.001**
Group [poor]	−0.13	−0.67–0.41	0.634
Syllable [consistent]	−0.33	−0.54–−0.12	**0.002**
Learning phase 2	−0.23	−0.42–−0.05	**0.014**
Learning phase 3	−0.42	−0.61–−0.24	**<0.001**
Group [poor] × Syllable [consistent]	−0.57	−0.88–−0.27	**<0.001**
Random Effects			
σ^2^	1.07		
τ_00 (Participant)_	0.31		
τ_00 (Non-word)_	0.04		
ICC	0.25		
N_Participant_	20		
N_Non-word_	6		
Observations	720		
Marginal R^2^/Conditional R^2^	0.117/0.338		

Note. AIC: 2166.8.
